# Association of APOE4 genotype and treatment with cognitive outcomes in breast cancer survivors over time

**DOI:** 10.1038/s41523-021-00327-4

**Published:** 2021-09-03

**Authors:** Kathleen Van Dyk, Catherine M. Crespi, Julienne E. Bower, Judith E. Carroll, Laura Petersen, Patricia A. Ganz

**Affiliations:** 1 Semel Institute and Department of Psychiatry and Biobehavioral Sciences, David Geffen School of Medicine, UCLA, Los Angeles, CA USA; 2 Jonsson Comprehensive Cancer Center, UCLA, Los Angeles, CA USA; 3 Department of Biostatistics, Fielding School of Public Health, UCLA, Los Angeles, CA USA; 4 Department of Psychology, UCLA, Los Angeles, CA USA; 5 Cousins Center for Psychoneuroimmunology, UCLA, Los Angeles, CA USA; 6 Department of Medicine (Hematology-Oncology), David Geffen School of Medicine, UCLA, Los Angeles, CA USA; 7 Department of Health Policy and Management, Fielding School of Public Health, UCLA, Los Angeles, CA USA

**Keywords:** Breast cancer, Signs and symptoms

## Abstract

This prospective longitudinal study of breast cancer survivors (*n* = 167) examined the association of apolipoprotein ε4 (APOE ε4) genotype with cognition and interactions with chemotherapy or endocrine therapy up to 6 years after treatment. In general, we found no effects of ε4 across timepoints and treatment exposures; post hoc analysis at 3–6 years suggested a trend towards worse cognition in the domains of attention and learning among ε4 carriers exposed to endocrine therapy. Further study is needed.

A significant proportion of breast cancer survivors experience disruptive and distressing cognitive difficulties after treatment^1^. There are mounting efforts to understand risk factors for cognitive dysfunction including genetic risk. A prime candidate is the apolipoprotein ε4 (APOE ε4) polymorphism; carriers of one or both APOE4 alleles are known to have increased risk for late-onset Alzheimer’s disease^2–4^. Initial studies of the effect of APOE ε4 on cognitive outcomes in breast cancer have been inconclusive. Subtle effects, if any, have been found across cross-sectional studies and prospective studies, which have, at most, 24 months of follow-up^3,5–8^ The potential interaction between APOE ε4 and long-term anti-estrogen endocrine therapies in breast cancer survivors is especially important given evidence of sex differences in the risk of APOE ε4-associated dementia and the potential influence of changes in hormonal functioning on dementia risk in aging women^9–11^.

This hypothesis-generating study aimed to examine the association of APOE ε4 status and treatment exposures with cognitive function in breast cancer survivors. This is a secondary exploratory analysis of the Mind Body Study, a prospective longitudinal study of the cognitive effects of endocrine therapy in breast cancer survivors, with follow-up for 3–6 years. We previously reported finding comparable performance on neuropsychological testing between breast cancer survivors exposed to endocrine therapy and those who were not, consistent across timepoints^12^. In this report, we examined differences in neuropsychological testing over timepoints (i.e., baseline, 6 months, 12 months, and 3–6 years) by APOE ε4 status, and interactions between APOE ε4 status and chemotherapy or endocrine therapy exposure.

The recruitment flow diagram is presented in Supplementary Fig. 1. Characteristics of the sample by APOE ε4 status are detailed in Table 1; there were no significant differences between APOE ε4 groups on any demographic or clinical variables. Across mixed-effects models, we did not see significant effects of APOE ε4 status in any cognitive domain nor any significant interactions of APOE ε4 × time, APOE ε4 × chemotherapy, or APOE ε4 × time × chemotherapy (*p*’s > 0.05; see Supplementary Table 1 and Supplementary Fig. 2). We also did not find the APOE ε4 × endocrine therapy or APOE ε4 × time × endocrine therapy terms to be significant (*p*’s > 0.05; see Supplementary Fig. 3). However, visualization of the pattern of cognitive scores over time stratified by APOE ε4 and endocrine therapy suggested an emergent change in APOE ε4 effects at the final timepoint among those who underwent endocrine therapy (Supplementary Fig. 3).

**Table 1:** Demographic and clinical characteristics of sample based on APOE4 status.

Baseline characteristic Mean (SD) or frequency (%)	Total *n* = 167	APOE4− *n* = 132	APOE4+ *n* = 35	*p*-Value
Age	51.2 (8.2)	50.9 (7.9)	52.2 (9.7)	0.40
IQ	113.8 (9.3)	114.1 (9.0)	112.5 (10.4)	0.36
Race				
White	131 (78%)	100 (76%)	31 (89%)	0.10
Non-White	36 (22%)	32 (24%)	4 (11%)	
Married				
No	61 (37%)	48 (36%)	13 (38%)	0.84
Yes	105 (63%)	84 (64%)	21 (62%)	
Education				
Less than college degree	32 (19%)	22 (17%)	10 (29%)	
College degree	51 (31%)	45 (34%)	6 (17%)	0.09
More than college degree	84 (50%)	65 (49%)	19 (54%)	
Employed full- or part-time				
No	60 (36%)	44 (33%)	16 (47%)	0.14
Yes	106 (64%)	88 (67%)	18 (53%)	
Income				
<$100,000	64 (39%)	51 (40%)	13 (38%)	0.89
≥$100,000	99 (61%)	78 (60%)	21 (62%)	
Surgery type				
Lumpectomy	109 (65%)	87 (66%)	22 (63%)	0.74
Mastectomy	58 (35%)	45 (34%)	13 (37%)	
Stage				
0	23 (14%)	18 (14%)	5 (14%)	0.39 (or 0.55 for stage 0/1 vs. 2/3)
1	75 (53%)	61 (46%)	14 (40%)	
2	53 (32%)	43 (33%)	10 (29%)	
3	16 (10%)	10 (8%)	6 (17%)	
Radiation—ever				
No	45 (27%)	38 (29%)	7 (20%)	0.30
Yes	122 (73%)	94 (71%)	28 (80%)	
Chemotherapy—ever				
No	79 (47%)	62 (47%)	17 (49%)	0.87
Yes	88 (53%)	70 (53%)	18 (51%)	
Anthracycline use (if ever had chemotherapy)				
No	65 (74%)	52 (74%)	13 (73%)	0.86
Yes	23 (26%)	18 (26%)	5 (28%)	
Endocrine therapy at 6 or 12 months				
No	55 (33%)	46 (35%)	9 (26%)	0.31
Yes	112 (67%)	86 (65%)	26 (74%)	

Given the rarity of the sample and ability to examine long-term effects, we conducted a focused post hoc analysis to probe cognitive function based on APOE ε4 status within the endocrine therapy-exposed subgroup at the final timepoint (i.e., 3–6 years after baseline). In this subgroup, there were *n* = 14 for the APOE ε4+ group and *n* = 51 for the APOE ε4− group. These univariate models included the covariates age, IQ, race, chemotherapy, and baseline domain score, and focused on testing the APOE ε4 term. Those carrying an APOE ε4 allele tended to exhibit worse cognition at this later timepoint with small to large effects particularly in the domains of Attention (*F*(1,57) = 5.05, *p* = 0.03, partial *η*^2^ = 0.08 and Learning (*F*(1,58) = 1.92, *p* = 0.17, partial η^2^ = 0.03) (see Fig. 1). Of note, 11/14 APOE4 carriers in this subgroup started endocrine therapy with an aromatase inhibitor and the majority continued on one through the final timepoint (*n* = 8).

**Fig. 1:**
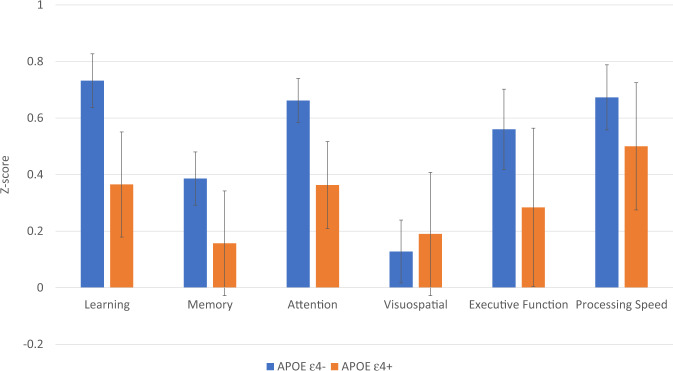
Cognitive domain scores in the endocrine therapy group by APOE ε4 status at 3–6 years post baseline. Models adjusted for age, IQ, chemotherapy, and race. Error bars represent ±1 SEM.

In summary, we did not find significantly worse cognitive function in breast cancer survivors with an APOE ε4 allele over time, nor any particular vulnerability in the presence of chemotherapy exposure or endocrine therapy across timepoints. However, we detected a small cognitive disadvantage specifically among APOE ε4 carriers exposed to endocrine therapy years after starting treatment on tests of learning and attention. Our results align with an emerging picture of APOE ε4 status and cognitive outcomes in cancer populations, which suggests small but meaningful interactive effects. For instance, others identified a link between APOE ε4 and poorer cognitive function, but only among those without a smoking history^13^ or exposed to chemotherapy^8^. The possibility that APOE ε4 status may interact with other risk factors is in line with the Alzheimer’s disease literature, where APOE ε4 status is not strictly determinative, but interacts or adds to the risks conferred by other factors^14^.

Our results also suggest a particular vulnerability among APOE ε4 carriers exposed to anti-estrogen endocrine therapy, although this must be interpreted with great caution given the small sample size. Despite equivocal evidence^9,12,15,16^, the effects of endocrine therapy warrant further, nuanced inquiry, given the close relationship between hormonal function and cognition in women^17^. The lifetime risk of developing Alzheimer’s disease in women is nearly twice that of men^18^ and the menopausal transition is a focus of understanding this discrepancy^19,20^. There is increasing evidence in dementia research that estrogen function interacts with APOE genotype^11,21^. Against this background, it is critical to fully understand the long-term effects of using pharmacological approaches to reduce available endogenous estrogen to treat hormone receptor-positive breast cancer. Further, the interaction of APOE ε4 status and endocrine therapy are likely both mild and latent if cancer-related cognitive decline and endocrine therapy exposure represent advanced cognitive aging^22^. There may be upstream processes that occur prior to cognitive symptoms, requiring longer observational periods to detect the effects of endocrine therapy and interaction with APOE ε4; this is the longest observational study of this nature to our knowledge and may be why other studies with shorter follow-up periods have not yet observed an interaction^8^.

This study has limitations. As APOE ε4 is present in only a minority of the population, our sample of APOE ε4+ breast cancer survivors is correspondingly small and precluded examining important sub-populations. We were also unable to look at the cumulative effects of chemotherapy plus endocrine therapy exposure. Our sample comprised largely White, high-functioning breast cancer survivors without cognitive impairment at study entry, and may not be representative of those who are older at initiation of endocrine therapy or have additional cognitive vulnerabilities that may put them at risk. In addition, we know from our prior study of this cohort that those who agreed to the final assessment may be more cognitively healthy^12^. These issues limit generalizability and power to detect effects and replication of our results in larger and more diverse samples is necessary, especially considering racial disparities in Alzheimer’s disease^23^.

Although our results provide reassurance that APOE ε4 status does not appear to play a significant role in dramatic cognitive changes in breast cancer survivors, late effects and interaction with endocrine therapy remain an important area of future hypothesis-driven research in both cognitively intact and cognitively vulnerable samples. Supporting cognitive health is a necessary component of supportive care in breast cancer survivorship including clarifying risk factors for impairment and risk for dementia in this growing population of older women.

## Methods

### Study design, sample, and measures

The Mind Body Study has been previously reported and the methods detailed^12,24,25^. In brief, between 2007 and 2011, we recruited newly diagnosed, early-stage breast cancer patients through clinical oncology practices and rapid case ascertainment using the Los Angeles County Surveillance, Epidemiology, and End Results Program registry with collaborating physicians and hospitals. Participants were 21–65 years of age, diagnosis of stage 0, I, II, or IIIA breast cancer, primary breast cancer treatments completed within the past 3 months; we excluded participants with prior cancer diagnosis or chemotherapy, or other cognitive risk factor (e.g., dementia, head trauma, epilepsy, etc.). The baseline visit occurred within three months of completing primary cancer treatment with surgery, radiation, and/or chemotherapy but before initiation of endocrine therapy if prescribed, with planned follow-up visits at 6 and 12 months after baseline (see Supplementary Fig. 4). At each study visit, we administered a questionnaire battery, collected blood, and administered a comprehensive neuropsychological battery, aggregated into norm-based domain *z*-scores: Learning, Memory, Attention, Visuospatial, Processing Speed, and Executive Function (test battery in Supplementary Table 2), higher scores indicating better performance. At the end of the 12-month visit, we re-consented participants for longer-term follow-up conducted ~3–6 years after initial diagnosis, depending on timing of study entry. Genomic DNA was extracted from peripheral blood leukocytes and assayed by real-time PCR using a TaqMan SNP genotyping assay (ThermoFisher Scientific). The UCLA Institutional Review Board approved the study and all participants signed informed consent.

### Analytic approach

The sample was grouped into APOE ε4 carriers (one or two alleles) and APOE ε4 non-carriers, consistent with other studies^8^, and treatment groups: chemotherapy (yes/no) and endocrine therapy (yes/no) based on whether or not they started endocrine therapy after baseline, modeled as time invariant similar to the intent-to-treat approach detailed in our prior analyses^12^. To test for group differences among cognitive domains over time, we fit linear mixed-effect models for repeated measures, which accommodates missing data. All models included random intercepts, and fixed effects included time (modeled as a categorical factor, i.e., baseline, 6 months, 12 months, and 3–6 years), age, IQ, and race. We examined the effects of APOE ε4, the effects of APOE ε4 by time, and the interaction of APOE ε4 by chemotherapy or endocrine therapy exposure, and time. Models testing endocrine therapy exposure included chemotherapy as a covariate. We used IBM SPSS v. 24 software and statistical significance was set at *p* < 0.05.

### Reporting summary

Further information on research design is available in the Nature Research Reporting Summary linked to this article.

## Supplementary information

Supplementary Information

Reporting Summary

## Data Availability

The data analyzed in this study are available upon reasonable request by email to the corresponding author in accordance with institutional policies.
