# Supplementary material for: Association of APOE4 genotype and treatment with cognitive outcomes in breast cancer survivors over time
Source: NPJ Breast Cancer. 2021 Sep 3;7:112. doi: 10.1038/s41523-021-00327-4 (PMC8417038; doi:10.1038/s41523-021-00327-4)
Supplement: Supplementary file 1 — Supplementary Information [file 41523_2021_327_MOESM1_ESM.pdf]

Supplementary Materials

Supplementary Figure 1: Recruitment Flow Diagram

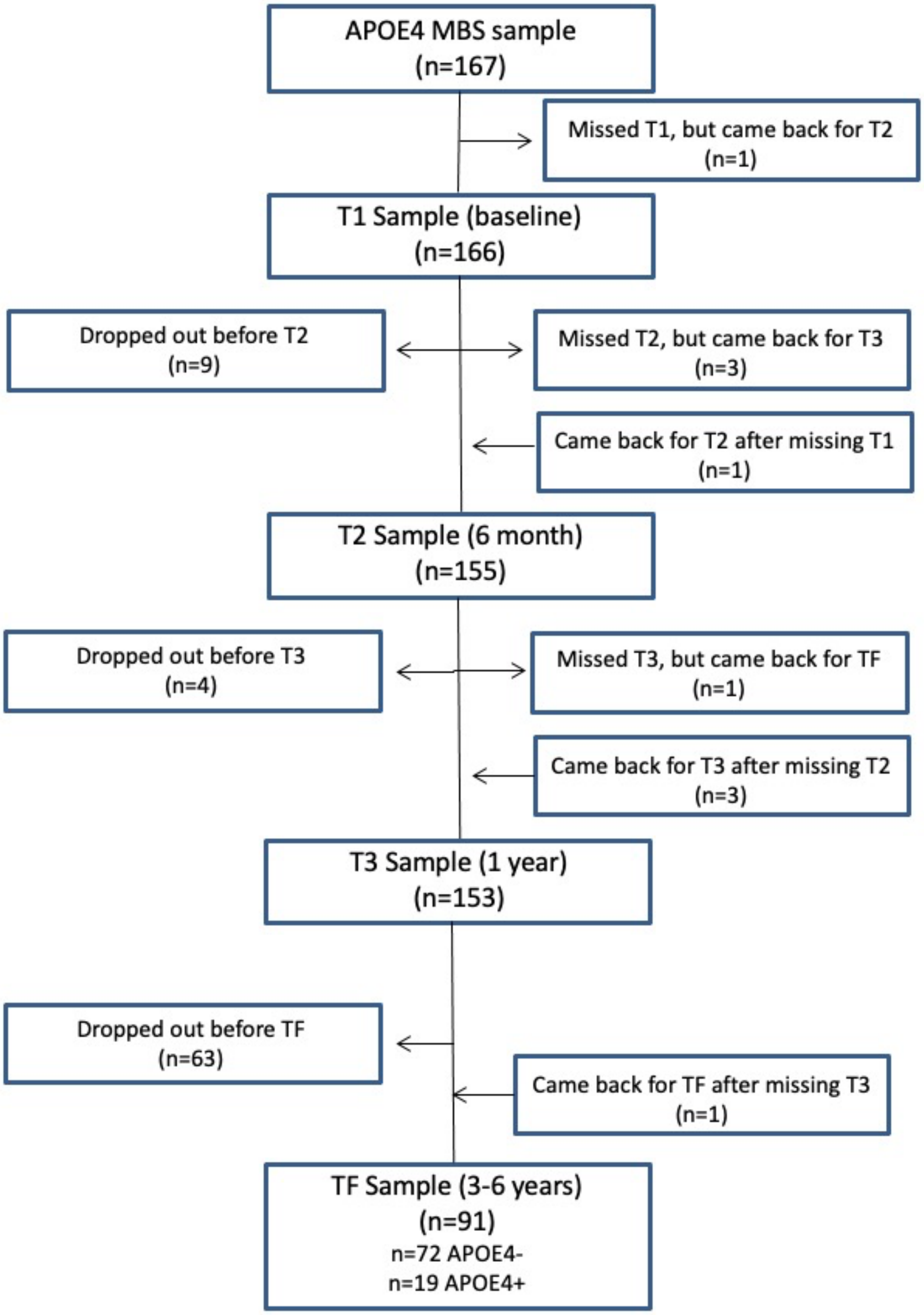

Supplementary Table 1: Estimates and F-Tests of Fixed Effects in Mixed Effects Models of Cognitive Domains*

	Learning		Memory		Attention		Visuospatial		Executive Function		Processing Speed	
APOE4 x Time		F(3, 186.78) = 1.66, p=.18		F(3, 187.06) = 0.29, p=.83		F(3, 184.41) = 0.1.20, p=.31		F(3, 208.28) = .75, p=.52		F(3, 145.09) = .16, p=.92		F(3, 159.64) = 0.56, p=.64
*APOE4 (ref=none) * Time at 3-6 yrs (ref=BL)*	0.11		0.06		0.21		-0.12		0.13		-0.01	
*APOE4 (ref=none) * Time at 12 mos (ref=BL)*	-0.14		-0.03		0.05		-0.10		0.02		-0.09	
*APOE4 (ref=none) * Time at 6 mos (ref=BL)*	-0.08		-0.05		0.06		-0.16		-0.01		0.001	
Chemo x APOE4	-2.6	F(1, 157.34) = 1.23, p=.27	-2.7	F(1, 156.75) = 1.53, p=.22	-0.001	F(1, 149.29) = .00, p=.99	-2.5	F(1, 150.32) = 1.42, p=.23	0.23	F(1, 157.75) = 0.89, p=.35	0.21	F(1, 156.66) = 0.80, p=.37
Chemo x APOE4 x Time		F(2, 161.41) = 0.14, p=.87		F(2, 154.34) = .80, p=.45		F(3, 181.15) = 1.61, p=.19		F(3, 204.44) = 0.916, p=.43		F(3, 141.95) = 0.16, p=.93		F(3, 159.14) = 1.26, p=.29
*APOE4 (ref=none) * Chemo (ref=no) * Time at 3-6 yrs (ref=BL)*	-0.01		-0.04		0.31		-0.31		0.03		-0.17	
*APOE4 (ref=none) * Chemo (ref=no) * Time at 12 mos (ref=BL)*	0.05		-0.15		-0.01		-0.13		0.08		-0.29	
*APOE4 (ref=none) * Chemo (ref=no) * Time at 6 mos (ref=BL)*	-0.04		0.01		-0.05		-0.10		0.09		-0.33	

ET x APOE4	-0.56	F(1, 158.71) = 4.17, p=.04	-0.48	F(1, 158.67) = 3.22, p=.07	-0.41	F(1, 150.62) = 3.13, p=.08	-0.46	F(1, 155.01) = 3.56, p=.06	-0.37	F(1, 158.69) = 1.56, p=.21	-0.42	F(1, 158.39) = 2.42, p=.12
ET x APOE4 x Time		F(3, 191.36) = 0.59, p=.62		F(3, 175.55) = 1.41, p=.24		F(3, 175.92) = 1.23, p=.30		F(3, 202.89) = 0.61, p=.61		F(2, 143.71) = 1.43, p=.24		F(3, 160.49) = .83, p=..48
*APOE4 (ref=none) * ET (ref=no) * Time at 3-6 yrs (ref=BL)*	-0.37		-0.34		-0.34		0.07		-0.85		0.02	
*APOE4 (ref=none) * ET (ref=no) * Time at 12 mos (ref=BL)*	-0.02		0.14		0.14		-0.18		-0.21		-0.08	
*APOE4 (ref=none) * ET (ref=no) * Time at 6 mos (ref=BL)*	0.02		0.15		0.15		-0.16		-0.16		0.07	

*Main effects and two-way interactions were examined separately in iterative models; models with three-way interactions included all subordinate terms. Data are assumed to be missing at random.

ET=endocrine therapy (no/yes); time, BL=baseline, 6-month, 12-month, and 3-year to 6-year visits (categorical). The fixed effects included in the models were age, intelligence quotient, chemotherapy exposure, and race, and all models included a random intercept.

Supplementary Figure 2: Cognitive Domain Scores by APOE4 status over time. Models adjusted for age, IQ, chemotherapy, and race.

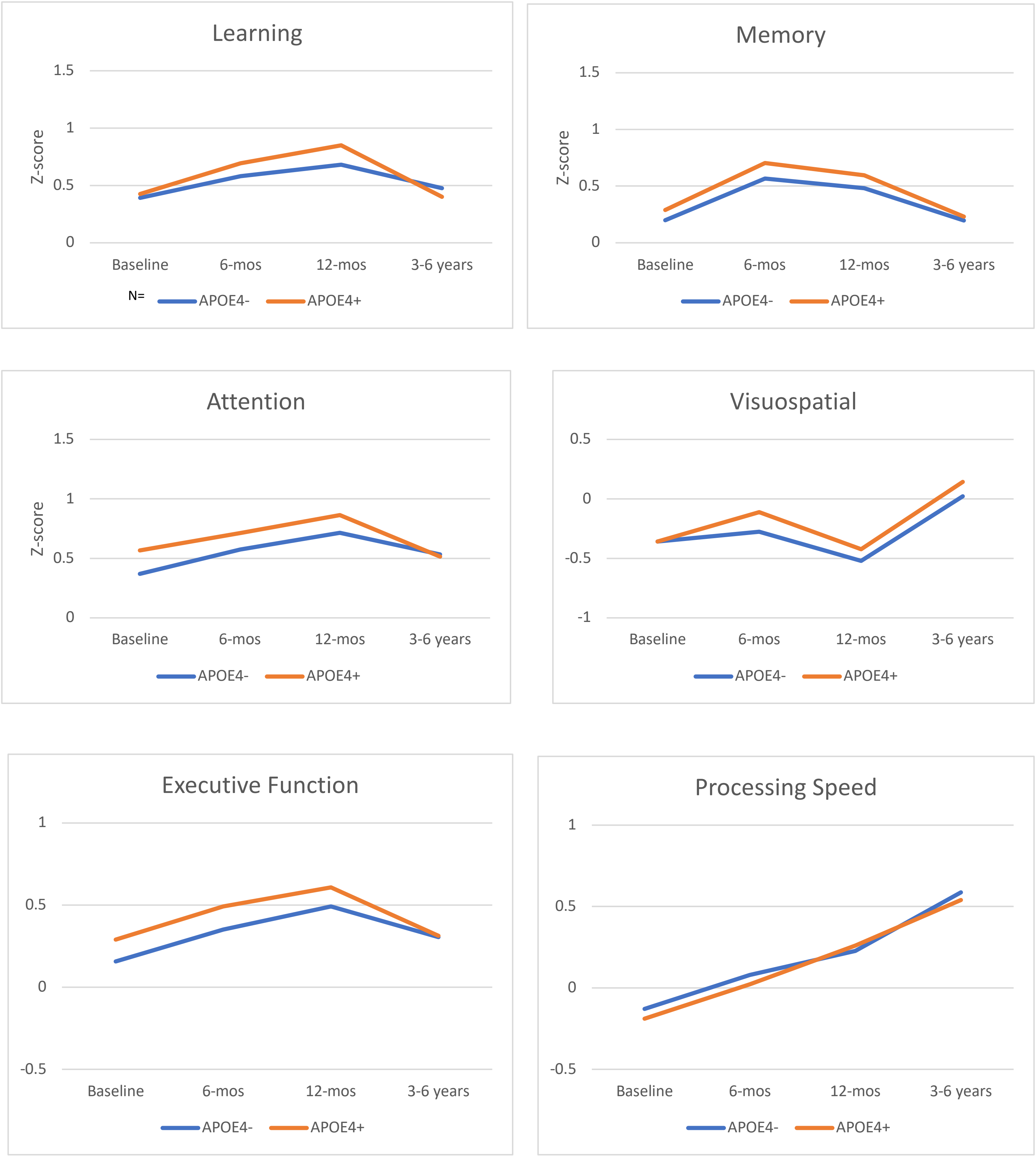

Data for Supplementary Figure 2

				95% Confidence Interval	
Learning		Mean	Std. Error	Lower Bound	Upper Bound
e4-	Baseline	0.392	0.057		
	6-mos	0.58	0.057	0.468	0.693
	12-mos	0.681	0.058	0.568	0.795
	3-6 years	0.477	0.079	0.322	0.632
e4+	Baseline	0.424	0.111	0.205	0.644
	6-mos	0.692	0.112	0.471	0.913
	12-mos	0.85	0.114	0.625	1.074
	3-6 years	0.401	0.151	0.104	0.699
Memory					
e4-	Baseline	0.198	0.055	0.089	0.306
	6-mos	0.567	0.054	0.461	0.673
	12-mos	0.48	0.052	0.378	0.582
	3-6 years	0.197	0.069	0.06	0.334
e4+	Baseline	0.288	0.108	0.076	0.501
	6-mos	0.704	0.105	0.497	0.912
	12-mos	0.597	0.102	0.395	0.799
	3-6 years	0.231	0.133	-0.031	0.493
Attention					
e4-	Baseline	0.37	0.049	0.273	0.467
	6-mos	0.574	0.047	0.482	0.666
	12-mos	0.715	0.045	0.627	0.803
	3-6 years	0.533	0.062	0.41	0.656
e4+	Baseline	0.566	0.097	0.375	0.756
	6-mos	0.713	0.09	0.534	0.891
	12-mos	0.864	0.088	0.691	1.038
	3-6 years	0.515	0.119	0.279	0.751
Visuospatial					
e4-	Baseline	-0.359	0.057	-0.471	-0.246
	6-mos	-0.275	0.057	-0.387	-0.164
	12-mos	-0.521	0.055	-0.63	-0.412
	3-6 years	0.022	0.081	-0.138	0.182
e4+	Baseline	-0.359	0.112	-0.58	-0.139
	6-mos	-0.112	0.11	-0.329	0.104

	12-mos	-0.423	0.109	-0.639	-0.207
	3-6 years	0.143	0.156	-0.165	0.451
Executive Function					
e4-	Baseline	0.157	0.058	0.042	0.272
	6-mos	0.351	0.06	0.233	0.468
	12-mos	0.492	0.059	0.376	0.609
	3-6 years	0.306	0.106	0.096	0.516
e4+	Baseline	0.29	0.114	0.065	0.516
	6-mos	0.493	0.117	0.263	0.723
	12-mos	0.608	0.117	0.377	0.839
	3-6 years	0.314	0.205	-0.091	0.719
Processing Speed					
e4-	Baseline	-0.129	0.06	-0.248	-0.01
	6-mos	0.081	0.056	-0.03	0.192
	12-mos	0.226	0.057	0.113	0.339
	3-6 years	0.587	0.104	0.382	0.792
e4+	Baseline	-0.189	0.119	-0.423	0.046
	6-mos	0.023	0.11	-0.195	0.241
	12-mos	0.261	0.113	0.037	0.484
	3-6 years	0.54	0.2	0.144	0.937

Supplementary Figure 3: Cognitive Domain scores by APOE4 status and endocrine therapy over time. Models adjusted for age, IQ, chemotherapy, and race.

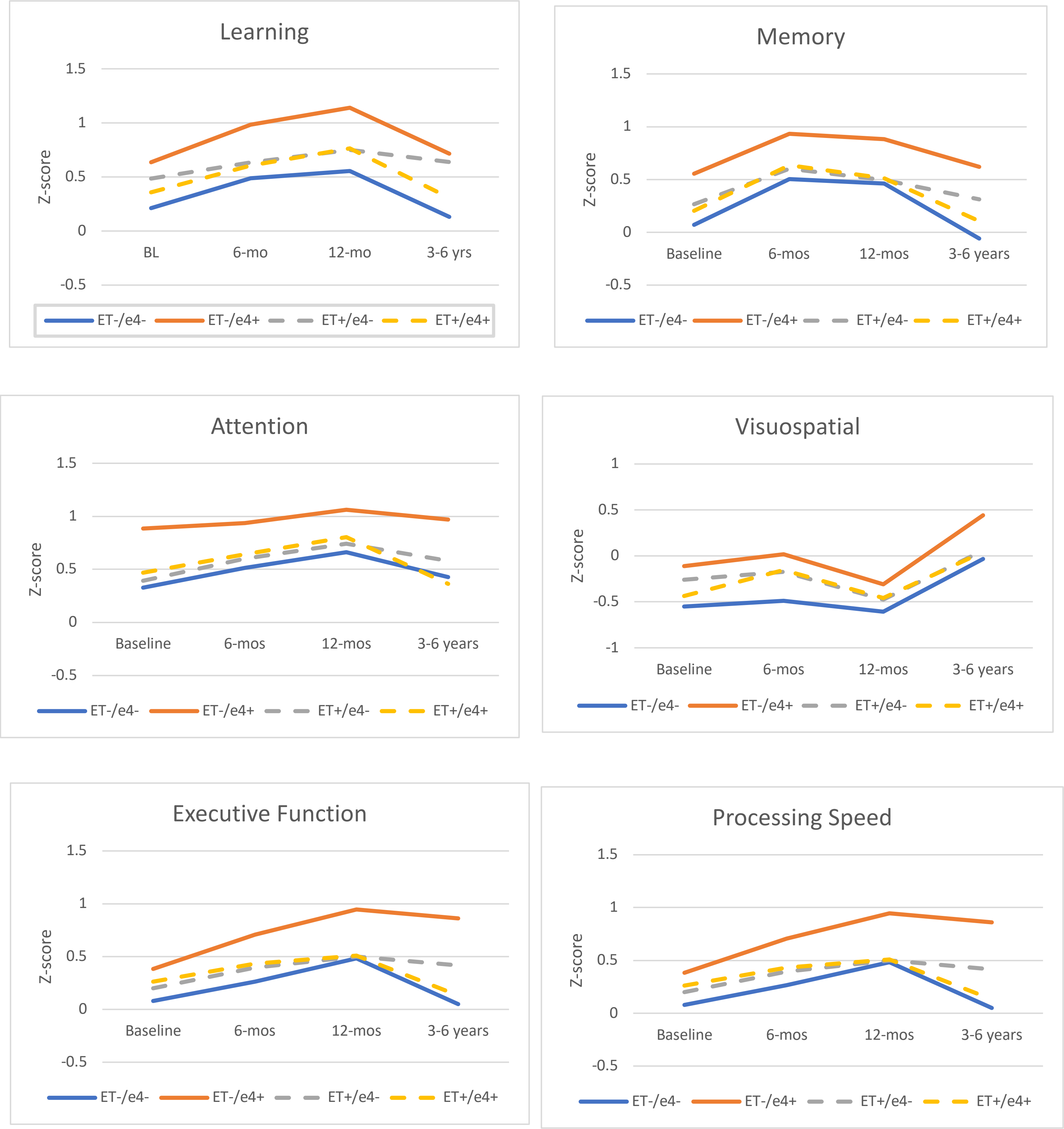

Data for Supplementary Figure 3

		Mean	Std. Error	95% Confidence Interval	
				Lower Bound	Upper Bound
Learning					
ET-/e4-	Baseline	0.21	0.10	0.03	0.40
	6-mos	0.49	0.10	0.29	0.68
	12-mos	0.56	0.10	0.36	0.75
	3-6 years	0.13	0.14	-0.14	0.40
ET+/e4-	Baseline	0.49	0.07	0.35	0.62
	6-mos	0.64	0.07	0.50	0.77
	12-mos	0.75	0.07	0.61	0.89
	3-6 years	0.64	0.09	0.46	0.82
ET-/e4+	Baseline	0.64	0.23	0.19	1.08
	6-mos	0.99	0.23	0.53	1.44
	12-mos	1.14	0.23	0.68	1.60
	3-6 years	0.72	0.29	0.14	1.29
ET+/e4+	Baseline	0.36	0.13	0.11	0.61
	6-mos	0.61	0.13	0.36	0.86
	12-mos	0.76	0.13	0.51	1.02
	3-6 years	0.31	0.17	-0.03	0.64
Memory					
ET-/e4-	Baseline	0.07	0.09	-0.11	0.25
	6-mos	0.50	0.10	0.32	0.69
	12-mos	0.46	0.09	0.29	0.64
	3-6 years	-0.06	0.12	-0.29	0.18
ET+/e4-	Baseline	0.27	0.07	0.13	0.40
	6-mos	0.60	0.07	0.47	0.73
	12-mos	0.50	0.06	0.37	0.62
	3-6 years	0.31	0.08	0.15	0.48
ET-/e4+	Baseline	0.56	0.22	0.12	0.99
	6-mos	0.93	0.22	0.50	1.37
	12-mos	0.88	0.21	0.47	1.30
	3-6 years	0.62	0.26	0.11	1.13
ET+/e4+	Baseline	0.21	0.12	-0.04	0.45
	6-mos	0.64	0.12	0.40	0.87
	12-mos	0.51	0.12	0.28	0.74

	3-6 years	0.11	0.15	-0.19	0.40
Attention					
ET-/e4-	Baseline	0.33	0.08	0.16	0.49
	6-mos	0.52	0.08	0.36	0.68
	12-mos	0.66	0.08	0.51	0.82
	3-6 years	0.43	0.11	0.21	0.64
ET+/e4-	Baseline	0.39	0.06	0.27	0.51
	6-mos	0.60	0.06	0.49	0.72
	12-mos	0.74	0.05	0.63	0.85
	3-6 years	0.58	0.07	0.44	0.73
ET-/e4+	Baseline	0.89	0.20	0.50	1.27
	6-mos	0.94	0.19	0.56	1.31
	12-mos	1.06	0.18	0.70	1.42
	3-6 years	0.97	0.24	0.50	1.43
ET+/e4+	Baseline	0.47	0.11	0.25	0.68
	6-mos	0.64	0.10	0.44	0.85
	12-mos	0.80	0.10	0.61	1.00
	3-6 years	0.36	0.14	0.09	0.63
Visuospatial					
ET-/e4-	Baseline	-0.55	0.10	-0.74	-0.36
	6-mos	-0.49	0.10	-0.69	-0.29
	12-mos	-0.61	0.10	-0.80	-0.42
	3-6 years	-0.03	0.15	-0.32	0.26
ET+/e4-	Baseline	-0.26	0.07	-0.40	-0.12
	6-mos	-0.17	0.07	-0.31	-0.04
	12-mos	-0.48	0.07	-0.61	-0.34
	3-6 years	0.06	0.10	-0.13	0.25
ET-/e4+	Baseline	-0.11	0.23	-0.56	0.34
	6-mos	0.02	0.23	-0.44	0.47
	12-mos	-0.31	0.23	-0.76	0.14
	3-6 years	0.44	0.31	-0.16	1.05
ET+/e4+	Baseline	-0.44	0.13	-0.69	-0.19
	6-mos	-0.15	0.12	-0.40	0.09
	12-mos	-0.46	0.12	-0.70	-0.21
	3-6 years	0.04	0.18	-0.32	0.40

Executive Function					
ET-/e4-	Baseline	0.08	0.10	-0.12	0.27
	6-mos	0.26	0.11	0.06	0.47
	12-mos	0.48	0.10	0.28	0.69
	3-6 years	0.05	0.19	-0.32	0.43
ET+/e4-	Baseline	0.20	0.07	0.06	0.34
	6-mos	0.39	0.07	0.25	0.54
	12-mos	0.50	0.07	0.36	0.64
	3-6 years	0.42	0.13	0.17	0.67
ET-/e4+	Baseline	0.38	0.23	-0.08	0.85
	6-mos	0.71	0.24	0.23	1.19
	12-mos	0.95	0.24	0.47	1.42
	3-6 years	0.86	0.40	0.07	1.65
ET+/e4+	Baseline	0.26	0.13	0.00	0.52
	6-mos	0.43	0.13	0.17	0.70
	12-mos	0.51	0.13	0.25	0.77
	3-6 years	0.13	0.24	-0.34	0.60
Processing Speed					
ET-/e4-	Baseline	-0.12	0.10	-0.32	0.09
	6-mos	0.02	0.10	-0.17	0.22
	12-mos	0.23	0.10	0.03	0.43
	3-6 years	0.59	0.19	0.21	0.96
ET+/e4-	Baseline	-0.13	0.08	-0.28	0.01
	6-mos	0.11	0.07	-0.03	0.24
	12-mos	0.22	0.07	0.08	0.36
	3-6 years	0.58	0.13	0.34	0.83
ET-/e4+	Baseline	0.04	0.24	-0.44	0.52
	6-mos	0.41	0.23	-0.05	0.86
	12-mos	0.54	0.24	0.07	1.01
	3-6 years	0.86	0.40	0.08	1.65
ET+/e4+	Baseline	-0.26	0.14	-0.53	0.01
	6-mos	-0.09	0.13	-0.33	0.16
	12-mos	0.18	0.13	-0.08	0.43
	3-6 years	0.44	0.23	-0.02	0.90

Supplementary Figure 4: Diagram of the study

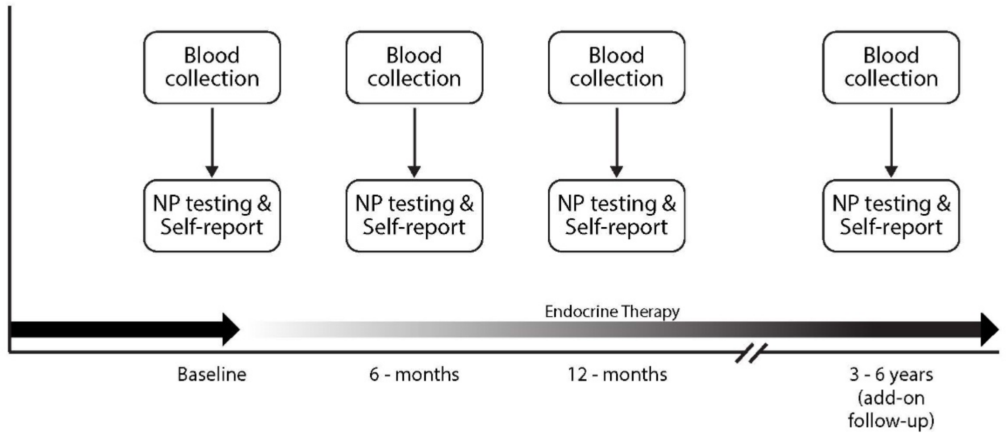

Supplementary Table 2: Neuropsychological Tests by Domain

Domain	Test/Measure
**Learning**	CVLT-II List A Total Trials 1-5<br>WMS-III LM I<br>BVMT-R Total Trials 1-3
**Memory**	CVLT-II List A Long Delay Free Recall<br>WMS-III LM II<br>BVMT-R Delayed Recall<br>ROCFT 3-minute Delayed Recall
**Attention**	WAIS-III Digit Span, Coding, Letter-Number Sequencing<br>TMT A completion time<br>PASAT Trial 2 total errors
**Visuospatial**	ROCFT copy<br>WAIS-III Block Design
**Executive Functioning**	TMT B<br>Stroop Color Word Interference[1]<br>Verbal Fluency (FAS)
**Processing Speed**	Grooved Pegboard[2]<br>Stroop Word Reading & Color Naming

BVMT-R=Brief Visuospatial Memory Test-Revised[3]; CVLT-II=California Verbal Learning Test-II[4];TMT=Trail Making Test[5]; ROCFT=Rey-Osterreith Complex Figure Test[6,7]; Paced Auditory Serial Attention Test[8]; WAIS-III=Wechsler Adult Intelligence Scale-3rd Edition[9]; WMS-III=Wechsler Memory Scale-3rd Edition[10]

Note: WAIS and WMS tests at BL, 6-, and 12-month visits are the 3rd edition; 4th edition tests were used at the 3-6 year visit, which are highly correlated to the 3rd edition tests. The Stroop test was not administered at the 3-6 year visit. Also of note, 20 participants were missing WAIS-III Digit Span data at BL.

1. Comalli PE, Wapner S, Werner H. Interference Effects of Stroop Color-Word Test in Childhood, Adulthood, and Aging. *J Genet Psychol*. 1962;100(1):47-53. doi:10.1080/00221325.1962.10533572
2. Klove H. Clinical Neuropsychology. *Med Clin North Am*. 1963;47:1647-1658. http://www.ncbi.nlm.nih.gov/entrez/query.fcgi?cmd=Retrieve&db=PubMed&dopt=Citation&list_uids=14078168.
3. Benedict RH. *Brief Visuospatial Memory Test - Revised*. Odessa, FL: Psychological Assessment Resources, Inc.; 1997.
4. Delis Kaplan, E., Kramer, J.H. DC, Delis DCKEKJH. California Verbal Learning Test-II. 2000.
5. Reitan RM. Validity of the Trail Making Test as an indicator of organic brain damage. *Percept Mot Skills*. 1958;8:271-276. doi:10.2466/pms.8.7.271-276
6. Strauss E, Sherman EMS, Spreen O. *A Compendium of Neuropsychological Tests*. 3rd ed. New York: Oxford University Press; 2006.
7. Mitrushina M, Boone KB, Razani J, D'Elia LF. *Handbook of Normative Data for*

Neuropsychological Assessment. Oxford University Press; 2005.
8. Gronwell DMA. Paced auditory serial-addition task: A measure of recovery from concussion. *Percept Mot Skills*. 1977;44:367-373.
9. Wechsler D. *WAIS-III, Wechsler Adult Intelligence Scale: Administration and Scoring Manual*. Psychological Corporation; 1997.
10. Wechsler D. Wechsler Memory Scale-Third Edition. 1997.
