# Supplementary material for: Association of APOE4 genotype and treatment with cognitive outcomes in breast cancer survivors over time
Source: NPJ Breast Cancer. 2021 Sep 3;7:112. doi: 10.1038/s41523-021-00327-4 (PMC8417038; doi:10.1038/s41523-021-00327-4)
Supplement: Supplementary file 2 — Reporting Summary [file 41523_2021_327_MOESM2_ESM.pdf]

Corresponding author(s): *Double-blind peer review submissions: write DBPR and your manuscript number here instead of author names.*

Last updated by author(s): *YYYY-MM-DD*

Reporting Summary

Nature Research wishes to improve the reproducibility of the work that we publish. This form provides structure for consistency and transparency in reporting. For further information on Nature Research policies, see our Editorial Policies and the Editorial Policy Checklist.

Statistics

For all statistical analyses, confirm that the following items are present in the figure legend, table legend, main text, or Methods section.

n/a	Confirmed	
☐	☒	The exact sample size (n) for each experimental group/condition, given as a discrete number and unit of measurement
☐	☒	A statement on whether measurements were taken from distinct samples or whether the same sample was measured repeatedly
☐	☒	The statistical test(s) used AND whether they are one- or two-sided<br>*Only common tests should be described solely by name; describe more complex techniques in the Methods section.*
☐	☒	A description of all covariates tested
☐	☒	A description of any assumptions or corrections, such as tests of normality and adjustment for multiple comparisons
☐	☒	A full description of the statistical parameters including central tendency (e.g. means) or other basic estimates (e.g. regression coefficient) AND variation (e.g. standard deviation) or associated estimates of uncertainty (e.g. confidence intervals)
☒	☐	For null hypothesis testing, the test statistic (e.g. F, t, r) with confidence intervals, effect sizes, degrees of freedom and P value noted<br>*Give P values as exact values whenever suitable.*
☒	☐	For Bayesian analysis, information on the choice of priors and Markov chain Monte Carlo settings
☒	☐	For hierarchical and complex designs, identification of the appropriate level for tests and full reporting of outcomes
☐	☒	Estimates of effect sizes (e.g. Cohen's d, Pearson's r), indicating how they were calculated

Our web collection on statistics for biologists contains articles on many of the points above.

Software and code

Policy information about availability of computer code

Data collection	Software was not used for data collection.
Data analysis	Data were analyzed using SPSS: IBM Corp. Released 2016. IBM SPSS Statistics for Windows, Version 24.0. Armonk, NY: IBM Corp.

For manuscripts utilizing custom algorithms or software that are central to the research but not yet described in published literature, software must be made available to editors and reviewers. We strongly encourage code deposition in a community repository (e.g. GitHub). See the Nature Research guidelines for submitting code & software for further information.

Data

Policy information about availability of data

All manuscripts must include a data availability statement. This statement should provide the following information, where applicable:

- Accession codes, unique identifiers, or web links for publicly available datasets
- A list of figures that have associated raw data
- A description of any restrictions on data availability

The data that support the findings of this study are available from the corresponding author upon reasonable request.

Field-specific reporting

Please select the one below that is the best fit for your research. If you are not sure, read the appropriate sections before making your selection.

☐ Life sciences ☒ Behavioural & social sciences ☐ Ecological, evolutionary & environmental sciences

For a reference copy of the document with all sections, see nature.com/documents/nr-reporting-summary-flat.pdf

Behavioural & social sciences study design

All studies must disclose on these points even when the disclosure is negative.

Study description	This is a prospective longitudinal observational study examining quantitative data
Research sample	Research participants were women breast cancer patients, representative of the study site's local demographics in southern California, who had recently completed primary cancer treatments but not yet begun endocrine therapy if indicated, in order naturalistically examine the impact of these treatments.
Sampling strategy	Participants were recruited through Rapid Case Ascertainment and this is a convenience sample.
Data collection	Data were collected via pen and paper cognitive tests and questionnaires administered in person by a research assistant and blood draw administered by local clinical research phlebotomist.
Timing	Data were collected at baseline, 6-month follow up, 12-month follow up, and an invited add-on evaluation conducted 3-6 years after baseline, depending on the date of study entry.
Data exclusions	Data were excluded for one participant for which there were concerns about data validity.
Non-participation	Nine participants dropped out after the first visit, 4 after the second visit, and 63 did not elect to participate in the last follow-up visit.
Randomization	Participants were no allocated to groups.

Reporting for specific materials, systems and methods

We require information from authors about some types of materials, experimental systems and methods used in many studies. Here, indicate whether each material, system or method listed is relevant to your study. If you are not sure if a list item applies to your research, read the appropriate section before selecting a response.

Materials & experimental systems

n/a	Involved in the study	
☒	☐	Antibodies
☒	☐	Eukaryotic cell lines
☒	☐	Palaeontology and archaeology
☒	☐	Animals and other organisms
☐	☒	Human research participants
☒	☐	Clinical data
☒	☐	Dual use research of concern

Methods

n/a	Involved in the study	
☒	☐	ChIP-seq
☒	☐	Flow cytometry
☒	☐	MRI-based neuroimaging

Human research participants

Policy information about studies involving human research participants

Population characteristics	Participants were on average 51 years old (SD 8.2), 35/167 were carriers of the APOE4 allele, 78% were white, and 81% had a college degree or higher.
Recruitment	Participants were recruited through Rapid Case Ascertainment using the Los Angeles County Surveillance, Epidemiology, and End Results Program registry with collaborating physicians and hospitals.
Ethics oversight	The UCLA Institutional Review Board approved this study.

Note that full information on the approval of the study protocol must also be provided in the manuscript.
